# Patient Safety Culture and Nurses' Turnover Intention: The Serial Mediating Roles of Burnout and Job Satisfaction

**DOI:** 10.1111/jocn.70304

**Published:** 2026-03-26

**Authors:** Seung Eun Lee, So Young Park, Philip Veliz, John Jardine

**Affiliations:** ^1^ College of Nursing, Yonsei University Seoul South Korea; ^2^ Department of Psychology Yonsei University Seoul South Korea; ^3^ School of Nursing University of Michigan Ann Arbor Michigan USA

**Keywords:** healthcare, intention to leave, nurses, organizational culture, safety culture, work environment

## Abstract

**Aim:**

To investigate whether patient safety culture is associated with nurses' turnover intention and to examine correlational sequential pathways involving burnout and job satisfaction, drawing on Conservation of Resources theory.

**Design:**

A descriptive, correlational design.

**Methods:**

This study used data collected during 2023 from a hospital‐wide patient safety culture survey conducted in four hospitals in South Korea. The sample comprised 3082 nurses from diverse units. Relationships among patient safety culture, burnout, job satisfaction, and turnover intention were examined using a mediation model within a structural equation modelling framework (WLSMV estimator with probit link), controlling for age and hospital tenure.

**Results:**

Patient safety culture was associated with lower burnout and higher job satisfaction. Burnout was associated with lower job satisfaction and with a higher likelihood of turnover intention, whereas job satisfaction was associated with a lower likelihood of turnover intention. When burnout and job satisfaction were considered together, the association between patient safety culture and turnover intention was explained through these two factors rather than by a direct pathway.

**Conclusion:**

Patient safety culture functions as an organizational resource that relates to reduced burnout and enhanced job satisfaction, which together relate to lower intention to leave.

**Implications for the Profession:**

Strengthening patient safety culture—alongside efforts to reduce strain and foster positive job attitudes—may support nurse well‐being and improve retention, thereby supporting continuity and safety of patient care.

**Impact:**

This study addresses persistent nurse turnover intention in hospitals and identifies patient safety culture as an organizational lever that operates through reduced burnout and improved job satisfaction. The findings can guide nurse leaders and policymakers in hospitals to implement culture‐focused strategies that support staff well‐being, enhance retention, and sustain safe patient care.

**Reporting Method:**

STROBE guidelines were followed.

**Patient or Public Contribution:**

No patient or public contribution.

## Summary

- What does this paper contribute to the wider global clinical community?
  ○ This study clarifies the mechanism through which patient safety culture reduces nurses' turnover intention, demonstrating that a stronger safety culture lowers burnout and enhances job satisfaction, which in turn decreases nurses' turnover intention.
  ○ These findings indicate that patient safety culture may serve as an organizational resource to support nurse well‐being and retention, offering practical considerations for leaders and policymakers while warranting adaptation and further evaluation in diverse settings.

## 1 Introduction

Patient safety culture is widely recognized as a central determinant of healthcare quality, shaping not only patient outcomes but also the well‐being and performance of frontline staff. Within hospitals, patient safety culture reflects shared values, beliefs, and norms that prioritize safety in everyday clinical work (Agency for Healthcare Research and Quality 2024a). Organizations with strong patient safety culture encourage open communication about errors, reduce blame for reporting mistakes, and foster preventive practices that enable early detection and mitigation of risks (Noor Arzahan et al. 2022). Such cultures typically support continuous organizational learning, effective teamwork and collaboration, empowering personnel at all levels to participate in safety improvement (Lee et al. 2023).

While most prior work has been focused on links between patient safety culture and clinical patient outcomes (Hajizadeh et al. 2025), emerging evidence suggests that patient safety culture is also associated with indicators of staff well‐being (Kim et al. 2023) and workforce stability (Sun et al. 2023). However, these associations have most often been examined separately rather than within a single analytic framework that accounts for their interrelationships. As a result, less is known about how patient safety culture relates to turnover intention when burnout and job satisfaction are considered simultaneously. Addressing this gap is timely in healthcare settings, where nurses' turnover intention remains a persistent and pressing challenge.

## 2 Background

Conservation of Resources (COR) theory (Hobfoll 1989) provides a useful lens for explaining how patient safety culture may be associated with staff well‐being and retention‐related intentions. The theory posits that individuals strive to obtain, protect, and retain valued resources (e.g., energy, time, self‐efficacy, supportive leadership, and positive organizational culture), and that actual loss—or threat of loss—of these resources is the core mechanism of stress, which could be related to elevated burnout. In this view, patient safety culture can be understood as organizational resource infrastructure that may help prevent resource loss and facilitate recovery and gain, thereby lowering burnout. Lower burnout may subsequently restore cognitive and affective capacity for positive job evaluations, which in turn support higher job satisfaction and lower turnover intention (Hobfoll et al. 2018). For example, workplace environments that support psychologically safe, non‐punitive responses to mistakes reduce anticipatory threat and impression‐management costs, preserving emotional and cognitive resources; this mitigates the exhaustion and cynicism that contribute to burnout (Mallick et al. 2024) while maintaining the attitudinal resources that underpin job satisfaction (Alsabhan et al. 2025). Also, team‐learning routines, clear protocols, and visible managerial support that bundle and retain informational, social, and affective resources increase predictability and control in clinical practice, while cultivating perceptions of resource gain, thereby reducing burnout, increasing job satisfaction, and ultimately decreasing turnover intention (Sinsky et al. 2021).

Previous research has shown that burnout is associated with lower job satisfaction among nurses (Lee et al. 2024; Maqbali et al. 2024). Exhaustion and disengagement diminish cognitive capacity, self‐regulation, and professional accomplishment, which depletes the attitudinal resources that sustain job satisfaction (Hu et al. 2025). A decrease in job satisfaction correlates with a higher turnover intention within healthcare environments (Gedik et al. 2023). Employees who perceive their work environments as safe and supportive report higher levels of satisfaction and exhibit a reduced likelihood of considering leaving their positions (Al‐Surimi et al. 2022). Yet, prior studies have typically examined these relationships in pairs—such as patient safety culture associated with outcomes (Al‐Surimi et al. 2022) or burnout associated with outcomes (Dai et al. 2025)—rather than specifying and testing how patient safety culture, as an organizational resource may interact with burnout and job satisfaction in an integrated framework. Therefore, it is essential to investigate these constructs within a comprehensive model to clarify their interrelationships and collective impact on turnover intention.

## 3 The Study

**FIGURE 1:**
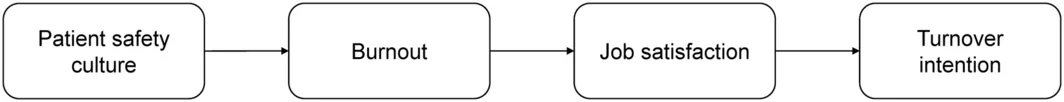
Hypothesized Serial Mediation Path Model.

This study aims to test a serial mediation model that traces a sequential pathway from patient safety culture to burnout to job satisfaction, and ultimately to turnover intention (Figure 1). Grounded in COR theory (Hobfoll 1989), we posit that patient safety culture is negatively associated with burnout, burnout is negatively associated with job satisfaction, and job satisfaction is negatively associated with turnover intention; we examine both indirect and serial indirect effects linking patient safety culture to turnover intention via burnout and job satisfaction. By specifying and estimating this COR‐consistent mechanism within a single model, we clarify how strengthening patient safety culture can promote nurses' well‐being and workforce retention.

## 4 Methods

### 4.1 Study Design

A descriptive, correlational, cross‐sectional study design was employed.

### 4.2 Study Setting and Recruitment

This study analysed data from a hospital‐wide patient safety culture survey conducted in 2023. The survey was implemented across four large, nonprofit, metropolitan teaching hospitals in South Korea, each exceeding 800 inpatient beds. These hospitals were purposively selected because they shared comparable organizational features—size, teaching status, nonprofit governance, and urban location—thereby minimizing contextual heterogeneity and enhancing the external relevance of findings (Hesgrove et al. 2024). All participating hospitals held the highest grade (“S”) in the national nurse staffing level classification system, which is based on overall nurse‐to‐patient ratios used to differentiate inpatient nursing fees; importantly, this metric does not directly capture the number of patients assigned to an individual nurse (Kim et al. 2024).

In alignment with AHRQ recommendations, each hospital conducts a standardized safety culture assessment on a biennial cycle (AHRQ 2024b). In 2023, the survey was distributed to employees across the four institutions and yielded 6060 completed questionnaires, corresponding to a mean response rate of 75.13%. For the present analysis, we restricted the sample to registered nurses who provided direct care to patients. Data from nurse managers were excluded because their roles and experiences differ from those of staff nurses, potentially leading to different perceptions of safety culture. Among 6060 respondents across all roles, 3502 were registered nurses, of whom 3082 responses met our inclusion criteria and were retained for analysis.

### 4.3 Measures

Patient safety culture was assessed with the Korean adaptation of the Hospital Survey on Patient Safety Culture (HSOPSC) 2.0, which has been validated in Korean nursing samples and shows strong psychometric performance (Lee and Dahinten 2021). An example item is, “The actions of hospital management show that patient safety is a top priority.” Items were rated on five‐point Likert scales that followed the original HSOPSC 2.0 format, using either agreement anchors (1 = *strongly disagree* to 5 = *strongly agree*) or frequency anchors (1 = *never* to 5 = *always*) as appropriate (Sorra et al. 2019). In the present study, patient safety culture was operationalized as a composite score by averaging responses across all items, representing an overall perception of safety culture. This approach is consistent with COR theory, which conceptualizes organizational contexts as resource reservoirs as perceived by individuals (Hobfoll 1989). Higher average scores indicated more favorable perceptions of patient safety culture. In this sample, the Cronbach's alpha coefficient was 0.91, consistent with previous Korean research findings (Lee and Dahinten 2021).

Burnout was measured with a single‐item indicator asking respondents to select their level of burnout on a five‐point scale ranging from 1 (*no symptoms of burnout*) to 5 (*completely burned out*). This item, originally introduced by Schmoldt et al. (1994), has undergone psychometric evaluation in comparison with the Maslach Burnout Inventory Emotional Exhaustion subscale, supporting its use as a single‐item measure of burnout (Dolan et al. 2015).

Job satisfaction was captured with a single item from the Workplace Safety Scale of the HSOPSC 2.0 (Agency for Healthcare Research and Quality 2024c). Participants rated their overall satisfaction with their current job on a five‐point Likert scale from 1 (*very dissatisfied*) to 5 (*very satisfied*). Meta‐analytic findings indicate substantial convergence between single‐item and multi‐item measures of overall job satisfaction, suggesting that single‐item measures can capture the underlying construct (Wanous et al. 1997).

Turnover intention was assessed with a single item from the Workplace Safety Scale of the HSOPSC 2.0 (Agency for Healthcare Research and Quality 2024c) asking whether respondents were considering leaving their current hospital within the next year. Response options included “no intention to leave” and four distinct reasons for contemplating departure. For analysis, responses were dichotomized, with 0 indicating no intention to leave and 1 indicating any intention to leave. Single‐item measures of turnover intention have been commonly used in nursing workforce research, including studies examining associations with burnout (de Cordova et al. 2022).

Age (coded as 1 = 20s, 2 = 30s, 3 = 40s, 4 = over 50) and hospital tenure (coded as 1 = less than one year, 2 = 1 year to 5 years, 3 = 6 years to 10 years, 4 = 11 years to 15 years, 5 = 16 years to 20 years, 6 = 20 years or longer) were included as control variables, given their expected associations with the outcome variables (Sun et al. 2023).

### 4.4 Data Analysis

Descriptive statistics and Spearman's rho correlations were computed in SPSS version 30.0 to summarize participant characteristics and bivariate associations among patient safety culture, burnout, job satisfaction, and turnover intention. To test the hypothesized relations, path analysis was conducted in Mplus version 8.11 using the weighted least squares mean and variance adjusted (WLSMV) estimator with a probit link, given the categorical nature of the endogenous variables (Muthén and Muthén 2017). Burnout and job satisfaction were modelled as ordered categorical (5‐point) variables with thresholds estimated; turnover intention was treated as dichotomous (0 = no intention to leave; 1 = intention to leave). Missing data were handled using pairwise present analysis under the WLSMV estimator. Coefficients for paths predicting categorical outcomes are reported as STDYX‐standardized probit regression coefficients. Standardized indirect effects were also estimated, and 95% confidence intervals (CIs) are reported for all estimates.

### 4.5 Ethical Considerations

This study was approved by the Institutional Review Board of Yonsei University Health System (#4‐2025‐1189). The data analysed were derived from the hospitals' routine, biennial safety culture assessments and were used for secondary analysis. Participation was voluntary, and completion of the questionnaire was taken as provision of informed consent. Before responding, staff were notified that participation was optional, that their identities would not be disclosed, and that confidentiality would be maintained. All records were de‐identified and stored securely to protect participant privacy.

## 5 Results

### 5.1 Participant Characteristics

As presented in Table 1, 95.2% of respondents were female. Nearly half were in their 20s (47.9%), followed by those in their 30s (33.0%). Most participants (88.6%) held a bachelor's degree or higher. In terms of tenure, 74.4% had less than 10 years in their current hospital, and 91.4% had less than 10 years of experience in the current unit.

**TABLE 1:** Summary of sample characteristics (*N* = 3082).

Characteristics	Category	*N*	*%*
Sex	Male	149	4.8
	Female	2930	95.2
Age	20s	1477	47.9
	30s	1018	33.0
	40s	464	15.1
	Over 50	123	4.0
Educational level	Associate's degree	235	11.4
	Bachelor's degree	1668	81.2
	Master's degree or higher	152	7.4
Hospital tenure	Less than 1 year	419	13.6
	1–5 years	1320	42.8
	6–10 years	554	18.0
	11–15 years	293	9.5
	16–20 years	228	7.4
	21 years or longer	268	8.7
Unit tenure	Less than 1 year	715	23.2
	1–5 years	1675	54.4
	6–10 years	426	13.8
	11–15 years	152	4.9
	16–20 years	61	2.0
	21 years or longer	51	1.7
Employment status	Permanent	2000	97.4
	Temporary	53	2.6
Weekly working hours	Less than 30 h	50	2.4
	30–40 h	972	47.4
	More than 40 h	1028	50.2

*Note:* Percentages are based on non‐missing responses for each variable. Missing data; Sex: *N* = 3 (0.1%), Educational level: *N* = 1027 (33.3%), Unit tenure: *N* = 2 (0.1%), Employment status: *N* = 1029 (33.4%), Weekly working hours: *N* = 1032 (33.5%).

### 5.2 Descriptive Statistics and Correlations of Study Variables

Table 2 presents the means, standard deviations, and bivariate correlations among the study variables. Patient safety culture was negatively correlated with burnout (*ρ* = −0.27, *p* < 0.001), positively correlated with job satisfaction (*ρ* = 0.39, *p* < 0.001), and associated with lower turnover intention (*ρ* = −0.21, *p* < 0.001). Burnout was negatively associated with job satisfaction (*ρ* = −0.41, *p* < 0.001) and associated with higher turnover intention (*ρ* = 0.37, *p* < 0.001). Job satisfaction was associated with lower turnover intention (*ρ* = −0.41, *p* < 0.001).

**TABLE 2:** Descriptive statistics and correlations of study variables.

Variables	Mean	SD	1	2	3
1. Patient safety culture	3.5	0.5			
2. Burnout	2.6	0.9	−0.27***		
3. Job satisfaction	3.1	0.8	0.39***	−0.41***	
4. Turnover intention (1 = yes, 0 = no)	—	—	−0.21***	0.37***	−0.41***

*Note:* SD = Standard Deviation; Turnover intention coded 1 = yes, 0 = no; prevalence = 44.3% (1364/3082); Correlations are Spearman's rho with pairwise deletion.

*** *p* < 0.001.

### 5.3 Serial Mediation Analysis

**FIGURE 2:**
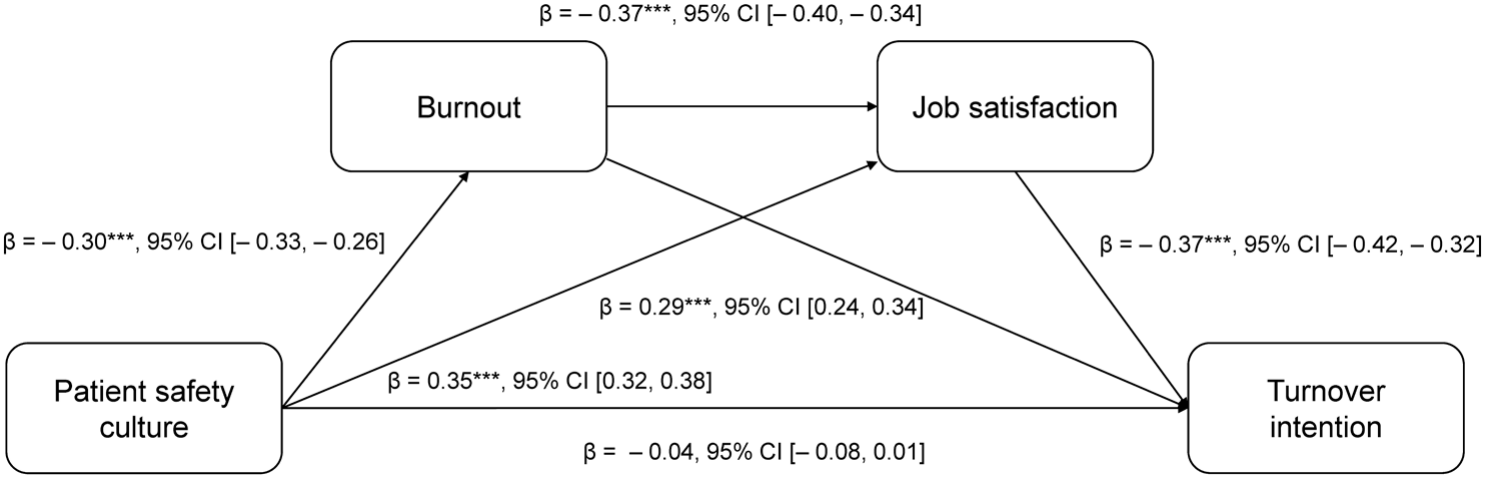
Multiple‐Mediator Path Model Including Both Parallel and Serial Paths (Patient safety culture→Burnout→Job Satisfaction→Turnover intention). Standardized probit regression coefficients are presented with 95% confidence intervals (CIs). Age and hospital tenure are included as covariates in the models of all endogenous variables. ***p < 0.001.

As shown in Figure 2, patient safety culture was associated with lower burnout (moderate effect; *β* = −0.30, *p* < 0.001, 95% CI [−0.33, −0.26]) and higher job satisfaction (moderate effect; *β* = 0.35, *p* < 0.001, 95% CI [0.32, 0.38]). Burnout was associated with lower job satisfaction (moderate effect; *β* = −0.37, *p* < 0.001, 95% CI [−0.40, −0.34]) and higher turnover intention (moderate effect; *β* = 0.29, *p* < 0.001, 95% CI [0.24, 0.34]). Job satisfaction was associated with lower turnover intention (moderate effect; *β* = −0.37, *p* < 0.001, 95% CI [−0.42, −0.32]).

As presented in Table 3, all indirect effects on turnover intention were significant. Patient safety culture was associated with lower turnover intention through burnout (small effect; *β* = −0.085, *p* < 0.001, 95% CI [−0.102, −0.069]) and through job satisfaction (small effect; *β* = −0.128, *p* < 0.001, 95% CI [−0.148, −0.107]), and sequentially via burnout and then job satisfaction (small effect; *β* = −0.040, *p* < 0.001, 95% CI [−0.049, −0.032]). The direct effect of patient safety culture on turnover intention (*β* = −0.035, *p* = 0.138, 95% CI [−0.080, 0.011]) was not significant, consistent with indirect‐only mediation.

**TABLE 3:** Direct and indirect effects of patient safety culture on turnover intention.

Paths	*β*	SE	95% CI	
			Lower	Upper
Patient safety culture→turnover intention				
Direct effect	−0.035	0.023	−0.080	0.011
Indirect effects				
Via burnout	−0.085***	0.009	−0.102	−0.069
Via job satisfaction	−0.128***	0.011	−0.148	−0.107
Via burnout and job satisfaction	−0.040***	0.004	−0.049	−0.032
Total indirect	−0.253***	0.013	−0.280	−0.227
Total effect	−0.288***	0.021	−0.329	−0.246

*Note:* *β* = standardized probit regression coefficient; SE = standard error; CI = confidence interval.

*** *p* < 0.001.

## 6 Discussion

This study examined the associations of patient safety culture on nurses' burnout, job satisfaction, and turnover intention using a serial mediation model. Our results showed that stronger patient safety culture was associated with lower burnout, lower burnout was associated with higher job satisfaction, and higher job satisfaction was related to lower turnover intention. Although cross‐sectional, this pattern positions patient safety culture as a potential organizational resource for nurse well‐being and workforce stability.

The inverse association between patient safety culture and burnout is consistent with prior research (Kim et al. 2023), with findings indicating that supportive safety environments are linked to lower psychological strain among nurses. Work settings with open, non‐punitive communication about error, an emphasis on learning rather than blame, and visible managerial support reduce anticipatory threat and cognitive load (Collins 2020), which is likely to lessen burnout. According to COR theory (Hobfoll 1989), such practices prevent resource loss and make needed resources, such as clear information, peer support, and reliable routines, more accessible in both routine and high‐pressure conditions. Our findings extend this evidence by specifying a resource‐based pathway through which a stronger safety culture relates to lower burnout (Kim et al. 2023).

Our results further support the well‐documented inverse relationship between burnout and job satisfaction. Exhaustion erodes cognitive abilities, self‐regulation, and a sense of professional achievement, thereby depleting the attitudinal resources that support job satisfaction (Hu et al. 2025). Consistent with earlier studies in nursing settings (Lee et al. 2024; Maqbali et al. 2024), we found that higher burnout was associated with lower job satisfaction in our sample. These results suggest that conserving resources at the strain stage may be crucial for maintaining positive work evaluations.

Consistent with previous organizational research (Gedik et al. 2023; Boamah et al. 2024), we found that lower job satisfaction was associated with higher turnover intention. Satisfaction reflects an overall appraisal of resource adequacy and fits; when satisfaction is lower, employees may seek to avert further losses by considering alternative roles or employers (Xie et al. 2024). This pattern is consistent with prior evidence identifying job satisfaction as a proximal predictor of nurses' intent to leave (Zhang et al. 2020).

Notably, the serial association from patient safety culture to turnover intention via burnout and job satisfaction was supported. Specifically, patient safety culture was indirectly related to lower turnover intention through its association with lower burnout and higher job satisfaction. The significant indirect effects indicate that the relationship between patient safety culture and turnover intention was accounted for by these mediating variables, rather than by a direct association. The direct association between patient safety culture and turnover intention was not statistically significant, suggesting an indirect‐only mediation pattern. This integrated correlational pattern provides insight into how an organizational resource may relate to more distal withdrawal cognitions via proximal strain and evaluative attitudes. Our finding aligns with the COR's perspective, which posits that resource‐conserving contexts interrupt resource loss spirals and facilitate resource gain (Hobfoll et al. 2018). Taken together, these results suggest that strengthening patient safety culture may enhance workforce stability by addressing these intervening factors.

### 6.1 Implications for Practice

Our findings suggest practical implications for hospital leaders. Efforts to strengthen patient safety culture may benefit from combining mutually reinforcing strategies, such as fostering open communication and psychological safety, providing visible managerial support, adopting non‐punitive responses to error that emphasize learning, standardizing patient hand‐offs to ensure reliable information sharing across transitions, and promoting collaborative teamwork to build shared situational awareness and mutual support (Membrillo‐Pillpe et al. 2023). In addition, leaders may consider implementing a psychologically safe reporting program, integrating patient safety culture metrics into routine monitoring and burnout prevention efforts, and using structured communication tools, such as check‐backs, to reinforce consistent team communication. Providing resilience training and peer support opportunities may further support nurses' recovery and resource replenishment in high‐demand clinical settings. Embedding these practices in everyday operations and linking them to feedback and learning systems may reduce burnout, enhance job satisfaction, and ultimately lower nurses' turnover intention.

### 6.2 Limitations

This study has several limitations. Although our cross‐sectional design allows assessment of associations, it does not support causal inference. Thus, serial mediation should be interpreted as correlational rather than as evidence of a causal process. Future research should employ multi‐wave longitudinal designs to establish temporal precedence. Our sample size was adequate for mediation analysis, but the non‐probability sample may limit the generalizability of the findings to organizations with different structures or cultural contexts. Also, all constructs were measured using self‐administered questionnaires, which may introduce self‐report and common‐method bias; future work could combine surveys with objective turnover data. The use of a single‐item measure is another limitation. This choice trades some precision and psychometric detail for lower respondent burden—an important feasibility consideration for hospital clinicians (Allen et al. 2022); future studies may consider supplementing with multi‐item scales. Finally, our sample came from four large metropolitan teaching hospitals that were intentionally similar to reduce cross‐hospital differences. Because the number of hospitals was small, clustering was not modelled; unmodeled clustering may bias standard errors. Future research with more sites should use cluster‐robust or multilevel approaches to examine between‐unit and between‐hospital variation.

## 7 Conclusions

Our findings indicate a correlational serial pathway linking patient safety culture to lower turnover intention via lower burnout and higher job satisfaction, while the direct patient safety culture–turnover intention link was not statistically significant, suggesting an indirect‐only pattern. This pattern is consistent with a resource‐based interpretation in which supportive, learning‐oriented, and psychologically safe work environments are associated with lower strain and more positive work evaluations. These findings suggest that strengthening patient safety culture may relate to improved workforce stability. Future studies should test these associations using multi‐wave, multi‐site designs that can establish temporal ordering and strengthen causality. From a clinical perspective, enhancing patient safety culture may represent a scalable organizational strategy to support nurse retention and workforce stability in high‐demand healthcare settings.

## Author Contributions

**Seung Eun Lee:** writing – original draft, writing – review and editing, conceptualization, data curation, funding acquisition, project administration, supervision. **So Young Park:** writing – original draft, writing – review and editing, methodology, investigation, formal analysis. **Philip Veliz:** writing – review and editing, methodology, formal analysis. **John Jardine:** writing – review and editing, methodology, formal analysis.

## Funding

This work was supported by the National Research Foundation of Korea (NRF) grants funded by the Korean government (MSIT) (RS‐2023‐00208138). The funding body did not influence the study design, data collection, analysis, interpretation, or the writing of the manuscript.

## Disclosure

Statistical Statement: We confirm alignment with the Journal's statistical guidelines; Philip Veliz and John Jardine served as the team statisticians.

## Conflicts of Interest

The authors declare no conflicts of interest.

## Supporting information


**Data S1:** STROBE Statement—Checklist of items that should be included in reports of cross‐sectional studies.

## Data Availability

The data that support the findings of this study are available on request from the corresponding author. The data are not publicly available due to privacy or ethical restrictions.
